# Comparison of Obturation Quality after MTA Orthograde Filling with Various Obturation Techniques

**DOI:** 10.3390/jcm10081719

**Published:** 2021-04-16

**Authors:** Hae Jin An, Hyunjung Yoon, Hoi In Jung, Dong-Hoon Shin, Minju Song

**Affiliations:** 1 Department of Conservative Dentistry, College of Dentistry, Dankook University, Cheonan 31116, Korea; ahj7793@naver.com (H.J.A.); macaco_co@naver.com (H.Y.); donyshin@dankook.ac.kr (D.-H.S.); 2 Department of Preventive Dentistry & Public Oral Health, College of Dentistry, Yonsei University, Seoul 03722, Korea; JUNGHOIIN@yuhs.ac; 3 Institute of Tissue Regeneration Engineering (ITREN), Dankook University, Cheonan 31116, Korea

**Keywords:** MTA orthograde filling, void, open porosity, Micro-CT, EZ-seal, OrthoMTA, hand condensation, compactor activation, reverse rotary motion, root filling technique

## Abstract

This study aimed to quantify and compare the obturation quality after mineral trioxide aggregate (MTA) orthograde fillings with three different obturation techniques. Thirty-three extracted human maxillary molars were collected. Distobuccal and palatal canals were prepared to an apical size of #40/06 with a Profile Ni-Ti system. All 66 canals were divided into two groups according to the material (EZ-seal or OrthoMTA) and then obturated using three different techniques: manual compaction using S-kondenser (group H), compactor activation (group C), or reverse rotary motion of Ni-Ti file (group R). The obturated roots were scanned using micro-computed tomography (micro-CT). The percentage of voids located in the apical 5 mm was measured separately, that is, closed, open, and total porosity. There was no relation between the filling material and obturation technique (*p* > 0.05). The percentage volume of open and total porosity was higher in EZ-seal than in OrthoMTA (open: *p* = 0.002, total: *p* = 0.001). Group H showed higher open and total porosity than groups C and R. Micro-CT analysis showed that the void volume after orthograde MTA fillings significantly decreased when the additional activation was accompanied by hand condensation. Obturation with a Ni-Ti file using reverse motion could be recommended as an MTA orthograde filling technique.

## 1. Introduction

Mineral trioxide aggregate (MTA) has been proven to have favorable physical, chemical, and biological properties [1,2,3]. It exhibits many profound properties that are not available in other traditional materials currently used in dentistry. It has proper radiopacity and dimensional stability and is less sensitive to moisture and blood contamination [4,5]. In addition, it provides superior sealing ability and biocompatibility, promoting biological repair and regeneration [6]. Originally, ProRoot MTA (Dentsply Tulsa Dental, Tulsa, OK, USA) was introduced to prevent communication between the root canal and periapical tissue. More so, it was used for perforation repair [7,8,9] and root-end filling after apical surgery [10,11]. Several studies have demonstrated favorable biological responses to the material [12,13,14]. Thus, its application has been expanded to vital pulp therapy such as pulp capping, pulpotomy, apexogenesis promoting apical barrier formation in teeth with immature apices, and regenerative endodontic therapy [15,16,17,18,19,20,21]. Recently, it was suggested as an orthograde root canal filling material [22]. OrthoMTA (BioMTA, Seoul, Korea), developed mainly for orthograde filling, was released into the market. OrthoMTA was shown to form an interfacial hydroxyapatite layer and release calcium ions, inducing the regeneration of the apical periodontium. Some studies have reported the superiority of MTA as an orthograde filling [23,24].

As a canal filling material, MTA has advantageous physical properties, such as sealing ability [25,26] and root reinforcement [27,28], compared to gutta-percha. However, some studies have reported that the sealing ability of MTA, when used for orthograde filling, was inferior to that of gutta-percha [29,30]. This was due to the difficulty in clinical manipulation, which is one of the major drawbacks of MTA [31]. MTA in the form of slurry paste is known to be difficult to handle and requires practice. It will be more difficult to avoid creating voids when obturating the narrow and confined root canal spaces. The presence of voids in the MTA filling might cause leakage from the canal space, leading to an unfavorable outcome.

To minimize the occurrence of voids, several obturation techniques have been suggested. These include the hand compaction technique using hand files and pluggers [22,32], the canal projection technique [33], the MTA pellet technique [34], delivery using a lentulo spiral [29], and ultrasonic activation [32,35,36,37,38]. In addition, the manufacturer of OrthoMTA suggested the use of a compactor as a way to improve the quality of orthograde obturation. A compactor, rotating at 60 rpm, moving up and down within 0.5 mm from the working length is recommended [24,26]. However, to date, a suitable technique for MTA obturation has not yet been established.

EZ-seal (Ezekiel, Taean, Korea) is a newly developed calcium silicate cement for orthograde filling of the entire root canal, and is known to be heavy metal-free and has a shorter setting time (1 h). Depending on the manufacturer, EZ-seal has a few advantages over the previous MTA. First, as a water-soluble natural polymer is added, the EZ-seal does not expand, and thus the risk of root fracture might decrease when obturating the canal. Second, it is easy to insert into the root canal as flowability is improved. In addition, the manufacturer claimed that MTA filling using Ni-Ti files with reverse rotary motion leads to a dense filling.

Therefore, this study aimed to quantify and compare the obturation quality after MTA orthograde fillings with three different obturation techniques: (1) manual compaction alone, (2) compactor activation, or (3) reverse rotary motion of Ni-Ti files, using micro-computed tomography (micro-CT) imaging. As the external void that exists between the material and dentin is more critical to sealing ability, the percentage volume of open porosity was mainly assessed. The null hypothesis is that the volume of voids within the apical 5 mm of the filled canals would be influenced by (1) the type of MTA and (2) the obturation technique.

## 2. Materials and Methods

### 2.1. Sample Selection

This study was approved by the Institutional Review Board Committee of Dankook Dental Hospital, Cheonan, Korea (DKUDH IRB 2020-07-001). The hopeless maxillary molars were extracted at the Department of Periodontology or Oral and Maxillofacial Surgery in Dankook Dental Hospital. All freshly extracted teeth were stored in a 6% sodium hypochlorite (NaOCl) solution (SENSE CLEANER, SNS Dental, Incheon, Korea) for 30 min and cleaned to remove debris, calculus, and granulation tissues. Radiographs were then taken to measure the canal curvature [39]. Thirty-three maxillary molars were collected as follows: (1) single canal in the distobuccal root and palatal root; (2) mature root with a closed apex; (3) no signs of caries, cracks, resorption, or perforation; and (4) canal of apical curvature between 0° and 20°.

**Figure 1:**
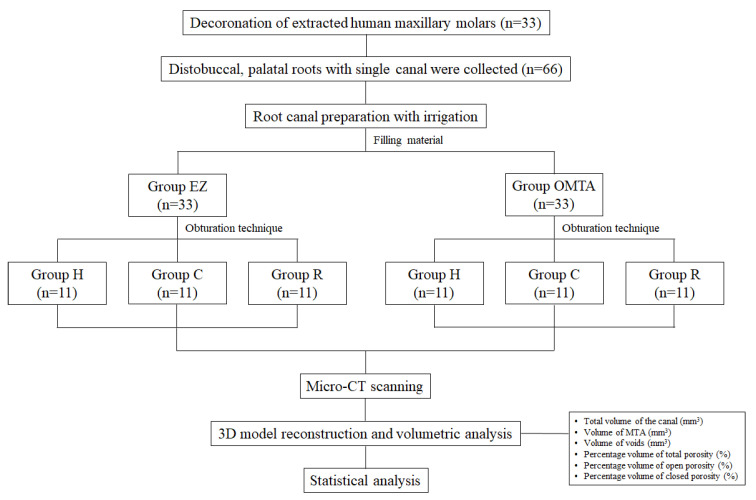
A flowchart of this study. EZ: EZ-Seal, OMTA: OrthoMTA, Group H: Hand condensation with S-Kondensor, Group C: Compactor activation + hand condensation with S-Kondensor, Group R: Reverse rotary motion of Ni-Ti file + hand condensation with S-Kondensor.

A total of 66 single canals from the distobuccal and palatal roots in one tooth were randomly divided into two groups (n = 33) according to the materials (EZ-seal or OrthoMTA, Table 1). Thirty-three canals in each material group were allocated to one of the following groups (n = 11) based on the obturation techniques: hand condensation with S-Kondensor (group H), compactor activation + hand condensation with S-Kondensor (group C), or reverse rotary motion of Ni-Ti file + hand condensation with S-Kondensor (group R). A flowchart of this study is shown in Figure 1.

### 2.2. Root Canal Preparation

The crowns were removed at the cementoenamel junction using a high-speed handpiece and bur under copious water spray, standardizing the length of the remaining root to 9 mm. The coronal canal enlargement was performed using Gates Glidden bur #2 and #3 (Dentsply Sirona, Ballaigues, Switzerland). A size 10 K-file (Dentsply Sirona, Ballaigues, Switzerland) was inserted into the canal until the tip of the file was visible at the apical foramen. The working length was determined by subtracting 1 mm from this length and confirmed from the radiographs taken. All root canals were instrumented with Profile Ni-Ti rotary files (Dentsply Maillefer, Ballaigues, Switzerland) until the #40/06 file reached the working length. Between each instrumentation step, patency was maintained by passing a size 10 K-file to the apical foramen. Each canal was irrigated with 1 mL of 6% NaOCl solution using a 30-gauge needle. Upon the completion of instrumentation, the canal was rinsed with 1 mL of 17% ethylenediaminetetraacetic acid (EDTA) for 1 min to remove the smear layer, followed by 5 mL of 6% NaOCl. All canals were dried with absorbent paper points (Millimeter-Marked Paper Points, Diadent, Cheongju, Korea).

### 2.3. Root Canal Obturation

OrthoMTA and EZ-seal were mixed according to the manufacturer’s instructions. According to the pilot study, the required amount of powder to obturate one canal was 30 mg for EZ-seal and 40 mg for OrthoMTA. To obtain the correct water/powder ratio, the powder was weighed using a digital electronic scale (CAS, Yangju, Korea). EZ-seal powder (30 mg) was mixed with 0.018 mL of saline to achieve a water/powder ratio of 0.6, and 40 mg of OrthoMTA powder and 0.012 mL of distilled water were mixed for 30 s to obtain a 0.3 water/powder ratio (Table 1).

Freshly mixed MTA was delivered into the prepared canal incrementally with an MTA carrier (BioMTA, Seoul, Korea). In group H, MTA was manually packed using S-Kondenser (Obtura Spartan, Earth City, MO, USA) until the entire root canal was filled, and the extra moisture on the compacted MTA was absorbed with paper points. In group C, after carrying MTA into the canal, a compactor (BioMTA, Seoul, Korea) with a #25/02 tip was inserted to the working length and rotated at 60 rpm with up and down motion. After obtaining an apical stop, S-Kondensor was used to compact the material as group H [23,24,26]. In group R, Profile size #40/06 was inserted into the canal after MTA delivery until it reached 1 mm within the working length, and rotated at 200 rpm in reverse rotary motion. After confirming the placement of the apical stop, the remaining portion was obturated with S-Kondensor as in group C. All teeth were stored at 37 °C with 100% humidity for 1 week to allow the complete setting of MTA. All canal preparations and obturations were performed by one endodontist (HJ An) to ensure consistency.

### 2.4. Micro-CT Evaluation

The filled roots were scanned using a high-resolution micro-CT scanner (Skyscan 1176, Bruker microCT, Kontich, Belgium) with a pixel size of 9 µm, using a 1 mm aluminum filter and a rotation step of 0.5°. Images obtained from the scanner were reconstructed with N-Recon (version 1.7.5.1, Bruker microCT, Kontich, Belgium) and aligned so that the long axis of the canal was perpendicular to the floor. Three-dimensional models were generated for volumetric analyses using CT An (version 1.19.4, Bruker, Kontich, Belgium) and CT Vox (version 3.3.0, Bruker, Kontich, Belgium).

The total canal volume, that is, the volume of interest (VOI), was set to 5 mm from the apical stop, which was generally 1 mm from the apex. The total canal volume consisted of MTA filling and void volume. Voids were divided into closed and open porosities. The void found inside the MTA filling was measured as closed porosity, and the void between the canal wall and MTA filling was measured as open porosity. The volume of porosity was calculated as a percentage of the total canal volume.

### 2.5. Statistical Analysis

Statistical analysis was performed using SPSS 21.0 (IBM SPSS Statistics, New York, NY, USA). The descriptive statistical analysis of the total canal volume and percentage volume of closed, open, and total porosity was expressed as mean and standard deviation (SD). A two-way analysis of variance (ANOVA) test was used to examine whether there was an interaction between two independent variables (material and technique). To analyze the differences between groups, a comparative analysis was performed by comparing the percentage volume of total, open, and closed porosity using the Student *t*-test, one-way ANOVA test, and Bonferroni’s post hoc test. For all tests, the significance level was set at *p* < 0.05. Before data analysis, extreme outliers were removed using data cleaning procedures.

As the open porosity between the material and dentin is a more critical factor for sealing ability, the percentage volume of open porosity was assessed to analyze the influence of filling material and obturation technique on void formation.

## 3. Results

**Figure 2:**
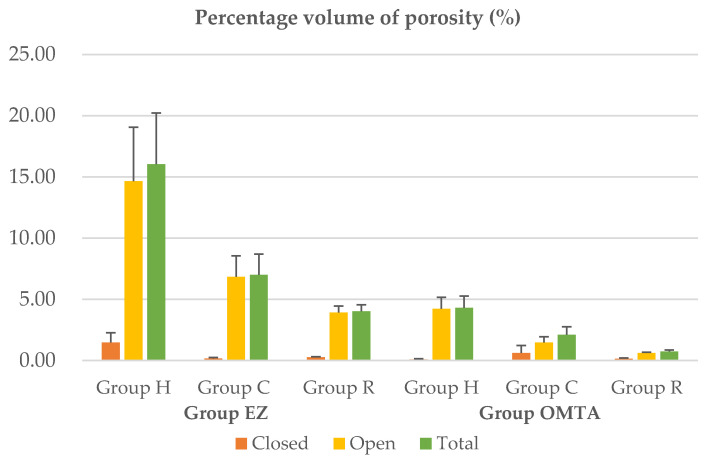
Percentage volume of closed, open and total porosity of each group.

The mean and standard deviation values of the total volume of the canal are presented in Table 2. The even volume of included canals from each group was verified (*p* = 0.133). The percentage volumes of closed, open, and total porosities are presented in Figure 2. Two-way ANOVA revealed no significant correlation between the filling material and obturation technique (Table 3).

When the materials were compared, group EZ(EZ-seal) exhibited a significantly higher percentage volume of open and total porosity than the OMTA(OrthoMTA) group (open, *p* = 0.002; total, *p* = 0.001). However, no significant difference was found in the percentage volume of closed porosity (*p* = 0.301, Table 4). Regarding the obturation technique, similar trends were observed in the two materials. Group H showed a significantly higher percentage volume of open and total porosity than groups C and R, whereas there was no significant difference between groups C and R (*p* = 0.423) (Table 5). Closed porosity did not differ among the three obturation techniques (*p* > 0.05, Table 5).

**Figure 3:**
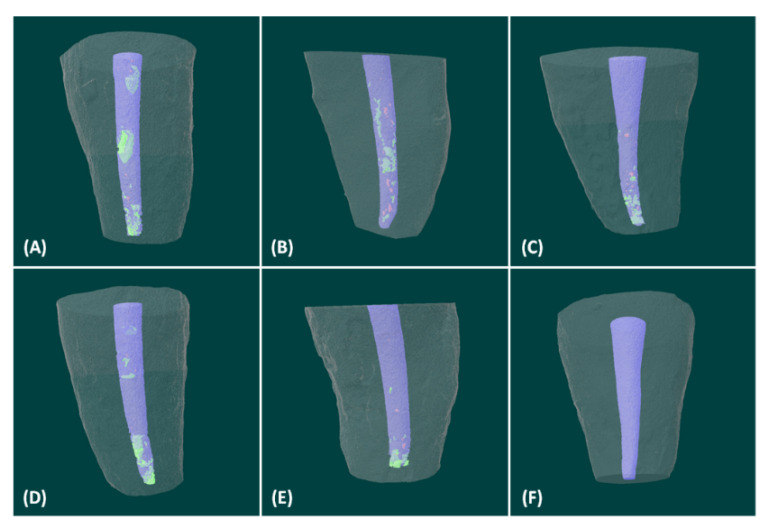
Representative 3-dimensional reconstructions of micro-CT scans illustrating the presence of distribution of voids within the root canal fillings. Red indicates closed porosity, green indicates open porosity, and purple indicates MTA fillings. (**A**) EZ-seal + Hand condensation (**B**) EZ-seal + Compactor activation (**C**) EZ-seal + Reverse rotary motion (**D**) OrthoMTA + Hand condensation (**E**) OrthoMTA + Compactor activation (**F**) OrthoMTA + Reverse rotary motion.

Representative 3D images illustrated the presence and distribution of voids within the root canal fillings, showing more open porosity (green) than closed porosity (red) in all groups (Figure 3). Most of them were distributed around the apical area but seemed to decrease in groups C and R with additional activation motion.

## 4. Discussion

The voids within filling materials may serve as hubs for microorganisms, leading to microleakage and long-term failure of endodontic treatment. The presence of voids can be influenced by several factors, such as the clinician’s experience, filling technique, root canal preparation technique, physical properties of the material, and the anatomical configuration of the root canal system [40]. In this study, to reduce the bias of morphologic factors, the maxillary molars were selected, since it was demonstrated that the distobuccal and palatal roots of maxillary molars contain only one canal [41,42,43,44,45]. In addition, all canal preparation and obturation techniques were performed by one operator in the same manner to ensure consistency.

In this study, micro-CT was used to quantify and compare the volume of voids within the canals. Scanning electron microscopy (SEM) has been used to analyze porosity and sealing ability. However, there were limitations, such as the destruction of the specimens for internal evaluation and two-dimensional analysis from the cross-sectional images. Artifacts and defects generated during specimen sectioning and preparation might act as a bias for accurate analysis, which might be critical for the results. Micro-CT has many advantages over SEM analysis. It is non-destructive and images can be analyzed in various planes of the sections. It provides a high-resolution image, and 3D visualization of the images allows the inspection of the internal structure as well. In addition, it requires less time and is less labor intensive [35,46,47,48,49,50,51,52,53,54,55,56,57,58]. Therefore, the micro-CT analysis was considered suitable for observing and quantifying the internal and external porosity from the canal space separately. The visualization of 3D images was also helpful in inspecting the distribution of porosity throughout the canal [26,35,39,49,50].

Most previous studies have investigated the volume and incidence of voids within canals without discriminating their locations. However, in this study, the percentage volumes of open and closed porosities were distinguished and measured separately. Open porosity, which is defined as the pores that occur at the interface between the MTA and dentin wall, may offer a pathway for microorganism growth and migration toward the periapical region [51,52]. Closed porosity is an isolated unfilled space within MTA, which has much less potential for bacterial growth and migration. Therefore, given the effect of each porosity and its clinical outcome, it is important to distinguish the location of the porosity. It seems rational to focus more on open porosity, which may contribute to apical periodontitis [50,53,54].

**Figure 4:**
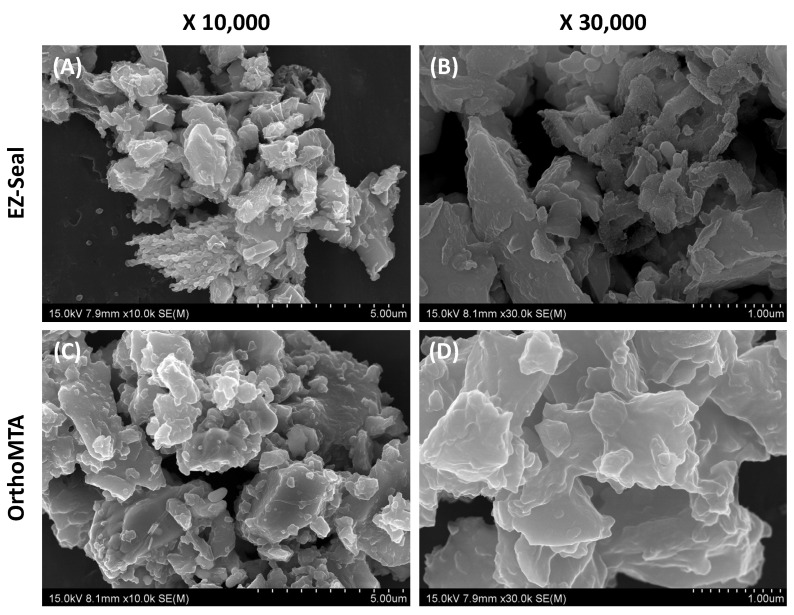
Scanning electronic microscopic (SEM) analysis of EZ-seal and OrthoMTA. EZ-Seal showed angular shapes and sharp points at low (**A**) and high (**B**) magnification. OrthoMTA showed homogeneous and circular at low (**C**) and high (**D**) magnification.

In the present study, the EZ group showed a significantly higher percentage volume of open porosity than the OMTA group. It is known that the handling of Portland cement is heavily dependent on particle size and shape [55]. To improve the filling operability of MTA, the particle size should be relatively uniform and small. Furthermore, there should be no coarse particles in the raw material and it should include spherical particles. Unlike OrthoMTA, the recently introduced EZ-seal had scarce information from the papers to support our results. To evaluate the particle characteristics of EZ-seal and OrthoMTA, we investigated the particles using SEM. From the SEM analysis, EZ-seal, which has large average particles, shows a wider distribution of particle size than OrthoMTA (Table 6). In addition, they are mixed types with particles having angular shapes and sharp points. They were less homogeneous and less circular (Figure 4). The better marginal adaptation between the canal wall and material in the OMTA group might be explained by its favorable characteristics with circular and homogeneous particle shapes.

This study showed a similar pattern of porosity volume according to the obturation techniques. The percentage volume of open and total porosity was significantly higher in group H than in groups C and R, whereas there was no difference between groups C and R. The vibration generated from the compactor activation and Ni-Ti file rotary motion might play an important role in obtaining dense MTA. The vibration produced a series of rapid compressive impulses that reduced the surface friction between the cement particles. When the mix becomes unstable, the individual particles begin to rotate, and the cement starts to flow. Consequently, the cement particles might be rearranged into a denser mass, and the unwanted entrapped air escapes to the surface [56]. Likewise, several studies have reported that activation during orthograde placement of MTA can result in denser root canal fillings [35,49,50]. Some studies have reported conflicting results [32,35], which can result from different materials and methods, for example, evaluation criteria such as open or total voids.

Even though there was no significant difference between groups C and R, group R showed less void volume compared to group C. This suggests the possibility of reverse motion of the Ni-Ti file as an obturation technique. The configuration of the Profile Ni-Ti file used in this study was designed to transport debris coronally [57]. Therefore, the reverse rotary motion of the profile during obturation might deliver MTA to the apical portion, improving the sealing ability. It might be more efficient to reduce the void from the canal walls compared to the compactor moving in the up and down directions. With this technique, operators can use the same file that is used for canal preparation, which is more efficient, convenient, and economical.

There are some limitations in the present study. First, we did not standardize the activation mode and condensation time for groups C and R. To avoid the injury of the teeth with uncontrolled excessive force/heat and provide a consistent result, it would be better to standardize and follow the procedure in detail. In addition, the Profile Ni-Ti rotary file, which is old generation and rarely used in clinic, was used in the present study. The Ni-Ti file system could affect the results on the obturation quality; therefore, further study would be required using various Ni-Ti file systems. Last, MTA orthograde filling has several drawbacks, such as discoloration [58,59,60], lower shear bond strength [61], and difficulty of removal [62]. Further studies dealing with those considerations are also needed to improve the clinical outcome of MTA orthograde filling.

## 5. Conclusions

Within the limitations of this study, micro-CT analysis showed that the void volume after orthograde MTA fillings was significantly decreased when the additional activation was accompanied by hand condensation. In particular, obturation using the reverse rotary motion of the Ni-Ti file showed the least open porosity, showing superior filling quality even though there was no significant difference from compactor activation. Therefore, obturation with a Ni-Ti file using reverse motion could be recommended as an MTA orthograde filling technique.

## Figures and Tables

**Table 1:** Root canal filling materials used in this study.

Group	Material	Water/Powder	Composition	Manufacturer
EZ	EZ-seal	0.6	Tricalcium silcate, Dicalcium silicate, Zirconium oxide	Ezekiel (Taean, Korea)
OMTA	OrthoMTA	0.3	Tricalcium silcate, Dicalcium silicate, Tricalcium aluminate, Bismuth oxide	BioMTA (Seoul, Korea)

**Table 2:** Total canal volume (mm^3^) in apical 5 mm of each group (Mean ± SD).

	Group EZ			Group OMTA			*p*
	Group H	Group C	Group R	Group H	Group C	Group R	
Total volume (mm^3^)	1.65 ± 0.40	1.61 ± 0.51	1.58 ± 0.22	1.74 ± 0.55	1.68 ± 0.26	2.09 ± 0.68	0.133
N	11	11	11	11	11	11	

**Table 3:** Two-way ANOVA considering filling material, obturation technique, and their interaction.

**(a) Total Porosity**					
**Source**	**Sum of Squares**	**df**	**Mean Square**	**F**	***p*-Value**
Corrected model	1538.710	5	307.742	6.748	0.000
Material	612.181	1	612.181	13.424	0.001
Obturation technique	606.881	2	303.441	6.654	0.003
Material * Obturation technique	190.024	2	95.012	2.083	0.135
Error	2280.105	50	45.602		
Corrected total	3818.815	55			
**(b) Open Porosity**					
**Source**	**Sum of Squares**	**df**	**Mean Square**	**F**	***p*-Value**
Corrected model	1312.172	5	262.434	5.297	0.001
Material	562.693	1	562.693	11.358	0.001
Obturation technique	519.537	2	259.769	5.243	0.009
Material * Obturation technique	125.419	2	62.710	1.266	0.291
Error	5633.255	50	49.543		
Corrected total	3789.337	59			
**(c) Closed Porosity**					
**Source**	**Sum of Squares**	**df**	**Mean Square**	**F**	***p*-Value**
Corrected model	14.781	5	2.956	1.510	0.204
Material	1.736	1	1.736	0.887	0.351
Obturation technique	3.221	2	1.611	0.823	0.445
Material * Obturation technique	8.619	2	4.309	2.201	0.121
Error	97.894	50	1.958	
Corrected total	112.675	55			

* Interaction between two factors.

**Table 4:** The percentage volume of porosity (%) in group EZ and OMTA.

	Group EZ	Group OMTA	*p*
Closed porosity (%)	0.68 ± 1.68	0.29 ± 1.07	0.301
Open porosity (%)	8.84 ± 10.22	2.16 ± 2.36	0.002
Total porosity (%)	9.43 ± 10.14	2.45 ± 2.49	0.001

**Table 5:** The percentage volume of porosity (%) in group H, C, and R.

	Group H	Group C	Group R
Closed porosity (%)	0.85 ± 2.05 ^a^	0.38 ± 1.25 ^a^	0.16 ± 0.39 ^a^
Open porosity (%)	9.97 ± 11.96 ^b^	4.31 ± 4.80 ^c^	2.36 ± 2.06 ^c^
Total porosity (%)	10.77 ± 11.81 ^d^	4.68 ± 4.75 ^e^	2.49 ± 2.04 ^e^

The same superscript lowercase letters in each row indicate no significant differences between obturation techniques (*p* > 0.05).

**Table 6:** Particle size (µm) of EZ-seal and OrthoMTA.

	Average Size (µm)	<90% (µm)	<50% (µm)	<10% (µm)
EZ-seal	3.75	7.86	2.88	0.29
OrthoMTA	2.62	4.64	2.36	1.18

## Data Availability

The data presented in this study are available on request from the corresponding author.
